# Potential application of lithium in Parkinson's and other neurodegenerative diseases

**DOI:** 10.3389/fnins.2015.00403

**Published:** 2015-10-27

**Authors:** Carol A. Lazzara, Yong-Hwan Kim

**Affiliations:** Department of Biological Sciences, Delaware State UniversityDover, DE, USA

**Keywords:** neuroprotection, calpain, GSK-3β, Bcl-2, and autophagy

## Abstract

Lithium, the long-standing hallmark treatment for bipolar disorder, has recently been identified as a potential neuroprotective agent in neurodegeneration. Here we focus on introducing numerous *in vitro* and *in vivo* studies that have shown lithium treatment to be efficacious in reducing oxidative stress and inflammation, increasing autophagy, inhibiting apoptosis, and decreasing the accumulation of α-synulcein, with an emphasis on Parkinson's disease. A number of biological pathways have been shown to be involved in causing these neuroprotective effects. The inhibition of GSK-3β has been the mechanism most studied; however, other modes of action include the regulation of apoptotic proteins and glutamate excitotoxicity as well as down-regulation of calpain. This review provides a framework of the neuroprotective effects of lithium in neurodegenerative diseases and the putative mechanisms by which lithium provides the protection. Lithium-only treatment may not be a suitable therapeutic option for neurodegenerative diseases due to inconsistent efficacy and potential side-effects, however, the use of low dose lithium in combination with other potential or existing therapeutic compounds may be a promising approach to reduce symptoms and disease progression in neurodegenerative diseases.

## Introduction

Lithium, introduced in 1949, is the most commonly used drug for the treatment of bipolar disorder, a chronic mental illness characterized by manic and depressive cycles. The efficacy of lithium in treating acute mania is long-established, and it is the standard against which other medications for bipolar disorder are measured (Young and Hammond, 2007). Meta-analysis of 14 randomized control samples showed that lithium, when used prophylactically, primarily reduced manic relapses, although its efficacy in reducing depressive relapses was significantly lower (Smith et al., 2007). Additional meta-analyses showed that lithium treatment reduced the number of suicides and suicide attempts in individuals with mood disorders (Cipriani et al., 2005; Baldessarini et al., 2006).

A growing body of evidence suggests that the benefits of lithium extend beyond mood stabilization. Lithium treatment has been shown to provide neuroprotection against neurological insults including excitotoxicity, ischemic damage, and traumatic brain injury (Basselin et al., 2006; Zhu et al., 2010). In addition, lithium has been shown to contribute to remyelination and axonal regeneration (Makoukji et al., 2012). In particular, lithium treatment has been associated with neuroprotection against neurodegenerative conditions such as Parkinson's, Alzheimer's, and Huntington's diseases as well as Amyotrophic Lateral Sclerosis (ALS). This review focuses on the effects of lithium on Parkinson's disease and some of the presumed mechanisms by which lithium provides its protective properties.

While studies suggest lithium can be an efficacious treatment for mood disorders and neurodegenerative conditions, there are several reports about lithium-induced neurotoxicity that, at its worst, is irreversible. High lithium doses are generally required for inducing neurotoxicity, however, it can occur at therapeutic dosages as well (Donaldson and Cuningham, 1983). The signs exhibited by the effected patients were mostly extrapyramidal in nature (Johnels et al., 1976; Kane et al., 1978; Ghadirian and Lehmann, 1980). There have also been reports of patients with cerebellar signs and Creutzfeldt-Jakob disease-like syndrome induced by lithium treatment (Smith and Kocen, 1988; Finelli, 1992). Research has shown, however, a correlation between lower doses of lithium and lower side-effects (Abou-Saleh and Coppen, 1989).

Parkinson's Disease (PD) is a basal ganglia disease, which associates with clinical motor symptoms such as bradykinesia, akinesia, resting tremor, muscular rigidity and postural instability, and non-motor symptoms of sleep disturbance, constipation, dysarthria, dysphonia, dysphagia, sialorrhoea, urinary incontinence and, “at the last, constant sleepiness with slight delirium” (Parkinson, 2002). Pathologically, PD is characterized by intracytoplasmic Lewy body inclusions and degeneration of dopaminergic neurons primarily in the substantia nigra pars compacta (SNc). Like other neurodegenerative diseases, the etiology of PD is largely unknown. Less than 10% of cases are probably caused by genetic mutations, most notably, in the gene encoding the presynaptic alpha-synuclein protein (Polymeropoulos et al., 1997). Some studies have demonstrated that prolonged occupational exposure to certain chemicals, particularly pesticides and heavy metals, such as Fe, Mn, Zn and Cu, is associated with an elevated risk of PD (Betarbet et al., 2000; Fukushima et al., 2013; Stelmashook et al., 2014).

## Lithium effects in Parkinson's disease

Diverse lithium studies in Parkinson's disease have been executed and included both *in vitro* and *in vivo* experiments. MPTP (1-methyl-4-phenyl-1,2,3,6-tetrahydropyridine) and 6-OHDA (6-hydroxydopamine) have been used to generate PD animal models and have been shown to activate the pro-apoptotic cysteine protease, caspase-3. In human neuroblastoma SH-SY5Y cells treated with MPP+ and in cultured rat cerebellar granule neurons treated with 6-OHDA, lithium inhibited the caspase-3 activation and prevented cell death (King et al., 2001; Chen et al., 2004). Endoplasmic reticulum (ER) stress, in combination with abnormal protein degradation has been implicated in the pathophysiology of PD as well as other neurodegenerative diseases (Lindholm et al., 2006). Lithium, via GSK inhibition, has been shown to protect cells from ER stress-induced lipid accumulation (Kim et al., 2005). In addition, chronic low-dose lithium exposure on SH-SY5Y cells demonstrated its neuroprotective effects, possibly mediated by regulating stress gene expression, stimulating glycolysis, accumulating extracellular pyruvate, and inducing resistance to oxidative stress due to nuclear factor erythroid 2-related factor 2 (NRF)-2 activation and miR-34a inhibition (Nciri et al., 2013; Alural et al., 2015). Similarly, lithium was found to be protective against oxidative stress in rat dopaminergic N27 cells which over-express A53T alpha-synuclein. In the brains of alpha-synuclein A53T over-expressing transgenic mice, lithium prevents/degrades paraquat/maneb-induced alpha-synuclein protein aggregation (Kim et al., 2011). Chronic lithium treatment *in vivo* and *in vitro* in the absence of either neurotoxin showed significant increases in tyrosine hydroxylase in the frontal cortex, hippocampus, and striatum of rats and mice as well as in human neuroblastoma SH-SY5Y and rat dopaminergic N27 cells (Chen et al., 1998; Lieu et al., 2014; Lazzara et al., 2015).

The combination of low-dose lithium and L-Dopa/Carbidopa (Sinemet®) in MPTP-lesioned mice has been shown to reduce MPTP-induced abnormal involuntary movements (AIMs) (Lazzara et al., 2015), while in another study using the MPTP-induced PD mouse model, combined administration of lithium and valproate improved motor function and increased the number of dopaminergic neurons in the substantia nigra, compared to controls. There was also a decrease in the concentration of the dopamine metabolite, dihydroxyphenyl acetic acid (DOPAC), in both the MPTP-treated and control groups; however, DOPAC loss was less severe in mice receiving the combined lithium-valproate treatment (Li et al., 2013).

Lithium has been considered as a potential apoptotic inhibitor. Studies in PD animal models have demonstrated that lithium can prevent MPTP-induced dopamine depletion and stimulate the up-regulation of B-cell lymphoma 2 (Bcl-2) and the down-regulation of Bcl-2–associated X protein (Bax) in the striatum (Youdim and Arraf, 2004). Over-expression of Bax hastens apoptotic cell death, which can be reduced by lithium's anti-apoptotic effect (Oltval et al., 1993). Bcl-2, on the other hand, is a proto-oncogene that is one of the key regulators of apoptosis. Over-expression of Bcl-2 provides protection against dopaminergic neurotoxins, by which lithium may reduce apoptosis (Offen et al., 1998).

## Putative modes of action of lithium

**Figure 1:**
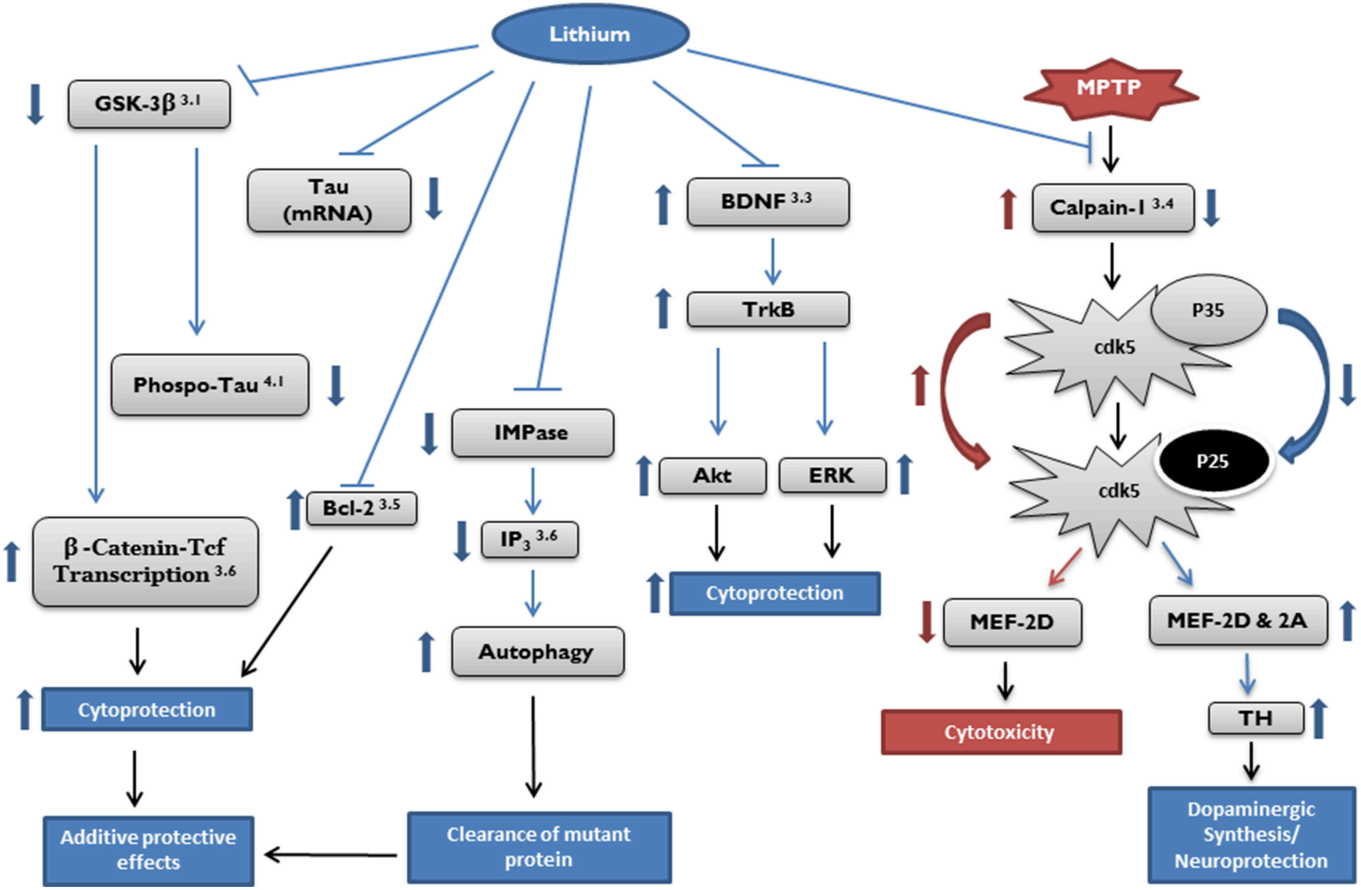
**Potential targets of cytoprotective effects by lithium**. Target pathways of lithium in neurodegenerative diseases described in the article are marked by section number (right top). Akt, protein kinase B; Bcl, B-cell lymphoma; BDNF, brain derived neurotrophic factor; Cdk, cyclin dependent kinase; ERK, extracellular signal-regulated kinase; GSK, glycogen synthase kinase; IMPase, inositol monophosphatase; IP_3,_ inositol 1,4,5-trisphosphate; MEF, myocyte enhancement factor; Tcf, T-cell factor; TH, tyrosine hydroxylase; TrkB, receptor tyrosine kinase B.

Numerous *in vitro* and *in vivo* studies over the last two decades have demonstrated that lithium provides neuroprotection, and as such, has the potential to become a therapeutic agent in the treatment of neurodegenerative diseases. Mechanisms of action of lithium include activating neurotrophic and neuroprotective cellular cascades, reducing oxidative stress, and decreasing apoptosis and inflammation. It has also been shown to enhance neurotrophic factors, reduce excitotoxicity, and provide mitochondrial stability (see Figure 1).

### Inhibition of GSK-3

Glycogen synthase kinase-3 (GSK-3) is a serine/threonine kinase that is involved in a number of intracellular signaling pathways. It exists in two isoforms, α and β. Dysfunction of the protein plays a role in the pathogenesis of both sporadic and familial forms of Alzheimer's disease. Up-regulation of GSK-3 activity leads to phosphorylation of the amyloid precursor protein (APP) and the protein tau, both of which are associated with the pathological processes that lead to the hallmarks of Alzheimer's disease (AD), amyloid-β plaques and neurofibrillary tangles (Hanger and Noble, 2011; Avila et al., 2012). Interestingly, in the MPTP mouse model, α-synuclein contributes to tau hyper-phosphorylation, an effect that is mediated by the activation of GSK-3β. This robust activation of GSK-3β was also observed in SH-SY5Y cells co-transfected with hDAT, mesencephalic neurons, striata of transgenic mice overexpressing α-synuclein, and in the postmortem striata of PD patients (Duka et al., 2009).

A number of researchers have demonstrated that lithium at concentrations of 1–2 mM inhibits GSK-3 (Bauer et al., 2003; Rowe and Chuang, 2004; Gould and Manji, 2005; Rowe et al., 2007). Lithium has been shown to reduce GSK-3 activity in two ways—both directly and indirectly—by increasing the inhibitory phosphorylation of GSK-3. The direct path by which GSK-3 is inhibited by lithium is via direct competition for a magnesium-binding site with GSK-3β (Jope, 1999). The indirect regulation of GSK-3 activity by lithium is through the activation of protein kinase B (also known as Akt). It is thought that the activation of Akt may provide additional neuroprotective effects downstream via modulation of forkhead box class O (FOXO), Bcl-2-associated death protein (Bad) (a pro-apoptotic protein of the Bcl-2 family), and murine double minute (MDM; Beaulieu et al., 2004, 2007; Avila and Hernández, 2007; Alural et al., 2015). Further, this inhibition of GSK-3 by lithium correlates with reduced tauopathy and degeneration *in vivo* (Hong et al., 1997; Noble et al., 2005). However, the lithium-induced GSK-3β regulation may be an acute down-regulation since chronic lithium exposure does not appear to have an impact on the level of GSK-3β expression nor its activity (Kim et al., 2011; Nciri et al., 2015).

### Inhibition of oxidative stress

Oxidative stress is believed to be one of the underlying causes of cellular dysfunction and death in PD. In PD patients, the cells in the SNpc exhibit increased levels of oxidative stress-induced damage in lipids, proteins, and DNA and decreased levels of glutathione (GSH) (Bosco et al., 2006; Nakabeppu et al., 2007; Zeevalk et al., 2008). The primary markers of oxidative stress include thiobarbituric acid reactive substances and regulation of several enzymes—superoxide dismutase (SOD), catalase (CAT) and glutathione peroxidase (Wang et al., 2003). When stress conditions are increased, SOD levels increase as well, leading to an elevated SOD/CAT ratio. An increase in oxidative stress is often linked to an increase in the cellular hydrogen peroxide concentration inducing lipid peroxidation in membranes, proteins and genes (Gsell et al., 1995).

In several *in vitro* studies, lithium administration was found to inhibit hydrogen peroxide-induced cell death as well as obstruct lipid peroxidation and protein oxidation in cortical cells (Shao et al., 2005; de Vasconcellos et al., 2006; Cui et al., 2007; Frey et al., 2007; Machado-Vieira et al., 2007; Kim et al., 2011). In addition, the ability of lithium to act as an anti-oxidant was ascribed to an increase in GSH levels in neurons, rat dopaminergic N27, and human SH-SY5Y neuroblastoma cells (de Vasconcellos et al., 2006; Kim et al., 2011).

### Activation of brain derived neurotrophic factor/receptor tyrosine kinase B

Brain derived neurotrophic factor (BDNF) helps to regulate neuronal and synaptic development and support the survival and plasticity of existing neurons (McAllister et al., 1999). The receptor tyrosine kinase B (TrkB) is activated by and facilitates the effects of the neurotrophins: BDNF, neurotrophin-3 (NT-3), and neurotrophin-4 (NT-4). These effects include neuronal differentiation and survival (Yoshii and Constantine-Paton, 2010).

Chronic lithium treatment was shown to significantly increase BDNF expression in the hippocampus as well as temporal and frontal cortices of rat brain; however, it was not accompanied by an increase in TrkB levels (Fukumoto et al., 2001). In a study which used primary cultures of rat cortical neurons, results with wild type and BDNF heterozygous and homozygous knock-outs showed that lithium application activates the BDNF/TrkB signaling pathway and protects neurons from glutamate excitotoxicity (Figure 1; Hashimoto et al., 2002). In two separate *in vitro* studies utilizing neural progenitor cells (NPCs), it has been suggested that lithium up-regulates BDNF production as evidenced by maximal cellular proliferation and neuronal differentiation (Su et al., 2007, 2009). It has been demonstrated that lithium increases mRNA of NGF, but not TrkA in rats, and inhibits TrkA-mediated signaling in PC12 cell cultures. In addition, NT-3 mRNA was decreased, but the mRNA of its receptor TrkC was increased by lithium (Burstein et al., 1985; Mudò et al., 1996). Although the cell proliferation effect by lithium in brain can be controversial, lithium-induced hippocampal neurogenesis in adult rodents has been reported by several groups (Fiorentini et al., 2010; O'Leary et al., 2012).

### Inhibition of the calpain—Cdk5 pathway

In the MPTP-induced PD mouse model, Smith et al. demonstrated a mechanism for dopaminergic neuronal loss which involves the downstream pathway of calpain-1 (2006). This mechanism includes modulation of the transcription factor myocyte enhancer factor 2 (MEF2) via cyclin dependent kinase 5 (Cdk5). A co-activator of Cdk5, p35, is converted to p25, a pathogenic form, by calpain-mediated cleavage of p35. Cdk5, bound to p25, becomes pro-apoptotic and leads to phosphorylation of MEF2 at Ser444, an inactivating site. The inactivation of MEF2 plays a critical role in dopaminergic cell loss (Smith et al., 2006). There has been no evidence that lithium inhibits calpain activity directly (Sasaki et al., 2006); however, it has been shown that lithium treatment robustly inhibits NMDA-receptor mediated calcium influx (Nonaka et al., 1998). As calpain activation is tied to calcium influx, it may be reasonable to speculate that lithium down-regulates calpain via this mechanism. We recently reported that lithium suppresses MPTP-induced calpain-1 expression and activity, which is very likely up-stream of MEF2 and tyrosine hydroxylase (TH) in the mouse brain. The efficacy of lithium for PD is, in part, derived from increased dopamine synthesis through TH-upregulation (Lazzara et al., 2015). The calpain-mediated Cdk5 pathway can be a known target for AD pathology as well. For example, the loss of regulation of Cdk5 has also been implicated in the formation of the pathological characteristics and the neurodegeneration associated with AD. An *in vivo* study using mice has revealed that the neurotoxic Cdk5 activator, p25, resulted in increased inflammation, deposition of amyloid and phosphorylated tau, and neuronal death. (Cruz and Tsai, 2004). The use of Cdk5 inhibitory peptide has been shown to reduce the effects of increased activation of Cdk5/p25 in mice, exhibiting decreased neuroinflammation, brain atrophy and cognitive decline (Sundaram et al., 2013). Lithium treatment in cultured cerebellar granule neurons prevents the increase of Cdk5/p35 fragmentation to Cdk5/p25 induced by colchicine (Jordà et al., 2005). In addition, treatment of cultured primary hippocampal neurons and rat striatum with lithium down-regulated calpain activity, Cdk5 activation, and cellular death induced by 3-nitropropionic acid (3-NPA) (Crespo-Biel et al., 2009).

It has been established that GSK3β is a key mediator of tau hyper-phosphorylation, and that lithium treatment inhibits GSK3β and consequently, tau hyperphosphorylation. Plattner et al. has demonstrated a connection between GSK-3β and Cdk5, showing that Cdk5 acts as a modulator of tau hyper-phosphorylation via the inhibitory regulation of GSK-3 (Plattner et al., 2006).

### Regulation of apoptotic proteins and glutamate excitotoxicity

Lithium has also been shown to influence levels of pro-apoptotic proteins. Bax, also known as Bcl-2–associated X protein, is a regulator that promotes apoptosis by binding to and antagonizing the Bcl-2 protein. The tumor suppressor protein, p53, targets both Bcl-2 and Bax and promotes growth arrest and cell death in response to cell damage (Basu and Haldar, 1998). In addition to being a major anti-apoptotic protein, Bcl-2 has been shown to induce regeneration of axons after injury (Huang et al., 2003).

A number of *in vitro* and *in vivo* experiments have demonstrated the neuroprotective effects of lithium attributed to increased Bcl-2 levels. Lithium treatment of cultured cerebellar granule cells stimulated an increase of mRNA and protein levels of Bcl-2; the Bcl-2/Bax protein level ratio increased by 5-fold after treatment for 5–7 days (Chen and Chuang, 1999). Lithium-induced increases in Bcl-2 expression were shown to cause neurogenesis in the hippocampus and entorhinal cortex in adult rodents as evidenced by an increase of axon diameters and improved neurite growth in the CA3 area of the hippocampus and an increase of myelination in the entorhinal cortex (Chen et al., 2000). In the MPTP-induced mouse PD model, a diet which included a high dose of lithium almost completely prevented the depletion of striatal dopamine and tyrosine hydroxylase, and the expected increase in dopamine turnover was prevented. Lithium was credited with providing neuroprotection by stimulating anti-apoptotic activity—increasing Bcl-2 level and reducing Bax (Youdim and Arraf, 2004). Phosphorylation of Bcl-2 at serine 70 is required for its complete anti-apoptotic function (Ruvolo et al., 2001), and Chen et al. have shown that lithium blocks induced apoptosis in mouse T hybridoma cells treated with ceramide and etoposide (2006). In this model, lithium inhibited Bcl-2 dephosphorylation and caspase-2 activation via reduction of protein phosphatase-2A activity (Chen et al., 2006). Changes in the expression of Bcl-2 and other pro-apoptotic genes have also been detected in human subjects taking lithium. The peripheral blood of patients with bipolar disorder was studied to observe changes in the gene expression profiles over time following treatment with lithium. This analysis identified the apoptotic pathway as the most affected by lithium, as after 1 month, those patients who responded positively to lithium treatment showed up-regulation of Bcl-2, while several pro-apoptotic genes, e.g.,: Bcl-2-antagonist/killer 1 (BAK1) and Bcl-2-associated agonist of cell death (BAD), were down-regulated (Lowthert et al., 2012).

Glutamate-induced excitotoxicity has been implicated in various neurodegenerative diseases including Huntington's disease, AD, and ALS, as well as in stroke, trauma and spinal cord injury (Friedlander, 2003; Lau and Tymianski, 2010). A number of studies have also associated glutamate-mediated excitotoxicity in the pathogenesis of PD. PARK2 is the E3 ubiquitin ligase parkin-encoding gene; its mutations cause PD. Mutations to the PARK2 gene can lead to an abnormally small parkin protein that is non-functional and is rapidly degraded. Parkin has also been shown to be involved in the function and stability of glutamatergic synapses. Further, the parkin mutations linked to PD trigger a proliferation of glutamatergic synapses with concomitant susceptibility to excitotoxicity (Helton et al., 2008).

Glutamate excitotoxicity has been shown to be associated with the up-regulation of Bax and p53, both of which are pro-apoptotic proteins, and the down-regulation of Bcl-2 (Chen and Chuang, 1999). The apoptosis attributed to glutamate was shown to be preceded by an increase in activator protein-1 (AP-1) caused by activation of c-Jun N-terminal kinase (JNK) and p38 mitogen-activated protein kinase (MAP kinase) and phosphorylation of c-Jun (Ser63) and p53 (Ser15) (Chen et al., 2003). In a study using cultured rat cerebellar granule cells, treatment with lithium prevented these signaling events and ameliorated the increase in apoptosis (Chi-Tso and Chuang, 2011).

### Other proposed pathways

There are a number of additional mechanisms by which lithium has been shown to act intracellularly. These include modification of cyclic adenosine monophosphate (cAMP)-mediated signal transduction (Jope, 1999; Gould et al., 2002; Einat et al., 2003); reduction in the arachidonic acid (AA) cascade (Chang et al., 1996, 2001; Chang and Jones, 1998; Rintala et al., 1999; Rapoport and Bosetti, 2002); negative regulation of the Smad3/4- transcription factor and protein levels of plasminogen activator inhibitor-1 (PAI-1); and induction of neurogenesis (Chen et al., 2000; Hashimoto et al., 2003). In addition, lithium has been shown to induce the survival pathway, MEK/ERK (Liang et al., 2008); increase levels of transcription factor β-catenin (Stambolic et al., 1996; Gould et al., 2004); and regulate autophagy via inositol inhibition and reduction of inositol 1, 4, 5-trisphosphate (IP3) levels (Sarkar et al., 2005; Sarkar and Rubinsztein, 2006, 2008; Fornai et al., 2008a; Klionsky et al., 2012).

## Lithium effects in other neurodegenerative diseases

### Alzheimer's disease

Abnormal levels of GSK-3 are associated with pathogenesis and neuronal death in individuals with AD (Bhat et al., 2004), and lithium has been shown to inhibit the GSK-3-related toxicity (Stambolic et al., 1996). In 1997, researchers demonstrated that lithium reduces phosphorylated tau *in vitro* and *in vivo* by inhibition of GSK-3 (Hong et al., 1997; Muñoz-Montaño et al., 1997). Lithium also prevented tau hyper-phosphorylation, thereby blocking its neurotoxicity and associated cell death (Alvarez et al., 1999). Further, lithium treatment resulted in a significant reduction of GSK-3 activity with concomitant decreases in the AD-associated tau phosphorylation, insoluble, aggregated tau accumulation, and axonal degeneration (Noble et al., 2005).

Various studies with AD animal models have shown that lithium can also provide beneficial effects. For example, aged double transgenic mice (AβPPSwe/PS1A246E) that display amyloid deposits were treated with lithium and showed attenuated γ-cleavage of amyloid precursor protein (APP) followed by reduction in amyloid-β plaque formation. These animals also showed improvement in spatial learning and memory abilities in addition to reduced autophagy activation (Zhang et al., 2010).

The promising studies showing GSK-3 inhibition by lithium have prompted many researchers to regard lithium as a potential therapeutic agent for the prevention and treatment of Alzheimer's disease. However, most clinical trials have provided ambiguous or inconsistent results. A case-control study using data from the General Practice Research Database in the UK showed that patients who received lithium treatment had a higher risk of diagnosis of dementia, which was believed to correlate to higher doses of the drug (Dunn et al., 2005). In a placebo-controlled, randomized, single-blind, multicenter study, lithium was given to 71 AD patients over a period of 10 weeks. CSF and plasma biomarkers (total tau, phosphorylated tau, Aβ42 and GSK-3) were monitored during that time period, and there was no significant difference in the biomarkers or in cognitive performance as compared to patients receiving a placebo (Hampel et al., 2009). However, in a 2007 study that compared elderly, bipolar patients (who are at a higher risk for dementia) who had received chronic lithium treatment, with bipolar patients who had not received lithium, it was shown that the lithium-treated patients had lower prevalence of dementia than the untreated group. In fact, the prevalence of the treated group was equivalent to the general, age-comparable population. The non-lithium-treated patients had an incidence of dementia that was six times greater (5% on lithium vs. 33% no lithium; Nunes et al., 2007). Although this study remains to be validated, it suggests lithium as a potential therapeutic for AD.

A recent, more promising, lithium study differed from previous ones in two ways: it was long-term (2 years) and involved patients who had mild cognitive impairment (MCI), not AD. The researchers measured the CSF biomarkers: Aβ42, phosphorylated tau and total tau, and assessed cognitive performance and drug safety. There was a significantly lower concentration of phosphorylated tau in the CSF of lithium-treated patients compared to those receiving a placebo, but there were no differences in the levels of Aβ42 and total tau. The lithium-treated group also had fewer conversions from mild cognitive impairment to AD, but this number was statistically insignificant. Nonetheless, the investigators believe their results show that lithium may slow the disease progression from cognitive impairment to dementia (Forlenza et al., 2011).

### Huntington's disease

In the 1970s, there were several clinical trials of lithium therapy for patients with HD. These trials included only small numbers of patients (9, 6, and 6) for very short time periods (6–12 weeks). In each case, the findings indicated that there were no improvements in involuntary movements, hyperkinesia, motor skills, or in the ability to perform everyday tasks, leading to the conclusion that lithium did not appear to be of therapeutic value in HD (Aminoff and Marshall, 1974; Leonard et al., 1975; Vestergaard et al., 1977). Since the 1970s, there have not been any reported human clinical trials utilizing lithium for treatment of HD; however, there has been a recent off-label use of lithium in a case study involving three individuals. Each of the patients received low doses of lithium and showed no further progression of chorea over periods of 2–4 years (Danivas et al., 2013).

Other studies showed that lithium treatment suppresses striatal lesions, reduces neurodegeneration, and stimulates cell proliferation in an excitotoxic rat model of HD. It has also been shown to reduce poly(Q) toxicity in cell models of HD; significantly improve motor performance (albeit with no improvement in longevity) in an HD mouse model; and protect against poly(Q)-mediated toxicity in a Drosophila model of HD (Wei et al., 2001; Carmichael et al., 2002; Wood and Morton, 2003; Senatorov et al., 2004; Berger et al., 2005). Two different studies looked at the results of combining lithium with a second mood-stabilizing drug to improve the efficacy of the treatment. Sarkar et al. showed that the treatment of an HD fly model with lithium in concert with rapamycin offered synergistic protection against neurodegeneration compared with either agent alone (Sarkar et al., 2008). Furthermore, there was better motor deficit improvement in two different mouse models of HD co-treated with lithium and valproate compared with monotherapy (Chiu et al., 2011).

### Amyotrophic lateral sclerosis

The protective effects of lithium in ALS were detailed in two papers (Fornai et al., 2008a,b). These include the normalization of the structure of altered mitochondria found in motor neurons as well as the removal of intracellular aggregates from motor neurons via increased autophagy; a stimulatory effect on mitochondrial biogenesis; inhibition of astroglial growth and proliferation; and neuronal differentiation. In a parallel study using human ALS patients and the G93A mouse (an ALS genetic animal model), researchers found that lithium provided significant neuroprotection. In the human trial, which lasted 15 months, the progression of the disease was significantly reduced in the lithium-treated group compared to the control group treated with riluzole for the same time period, while the mutant mice exhibited delayed onset of the disease and a longer life span (Fornai et al., 2008b). In another study with G93A mice in the same year, lithium in concert with valproic acid (VPA) was reported to delay the onset of ALS symptoms, increase life span, and the mice exhibited fewer neurological deficits as compared to mice treated with lithium or VPA alone (Feng et al., 2008). These results led the authors to suggest that lithium offers some promise as a treatment for human patients affected by ALS. In contrast, a subsequent multi-center consortium study reported that lithium treatment was not universally effective in ameliorating its symptoms in human patients (Chiò et al., 2010).

Based on the results from the Fornai et al. studies, a large 13-month phase II screening trial of lithium carbonate in ALS was undertaken in 2011. In contrast to earlier results, the researchers concluded that lithium carbonate does not slow the rate of decline of function in patients with ALS, as compared to a control group, and the lithium-treated patients showed no differences in quality of life and were more prone to adverse events (Miller et al., 2011). Around the same time, another randomized, double-blind, placebo-controlled trial showed similar negative results, and in fact, the trial was terminated prematurely because of futility (Aggarwal et al., 2010). These trials have demonstrated some safety concerns and the lack of promising therapeutic benefits from lithium treatment in ALS patients.

The inconsistent efficacy of lithium in treating ALS may be due to the fact that ALS is a complex disease with a constellation of cellular and molecular pathways involved in its pathophysiology. There has been a small subset of patients that demonstrated some improvement; however, it may be that lithium does not selectively target the pathways that would provide benefits to a majority of the cases. Another factor to consider is that there may be an optimal dose of lithium, which can be unique to each individual's disease stage, required to alleviate symptoms and prevent the pathology. However, most clinical trials were likely performed without identifying the optimal doses for patients. While some patients may show improvement with a standard dose, that dose in others may not impact on their pathophysiology or may create undesirable side-effects.

## Conclusions

Numerous *in vitro* and *in vivo* studies have shown that lithium provides potential therapeutic value in the prevention and/or treatment of neurodegenerative conditions. Multiple different biological mechanisms have been shown to contribute to these protective effects including the up-regulation of neuroprotective proteins including Bcl-2 and its actions on regulation of apoptosis and cellular resilience, such as GSK-3. Further clinical and experimental studies with lithium are needed to determine if the cellular and molecular biological properties of the drug can be a part of a therapeutic strategy for Parkinson's and other neurodegenerative diseases. Lithium-only treatment may not be a suitable therapeutic for neurodegenerative diseases due to inconsistent efficacy and potential side-effects, however, the use of low dose of lithium in combination with other potential or existing therapeutic compounds may be a promising prophylactic approach to reduce symptoms and disease progression in neurodegenerative diseases.

## Author contributions

CL wrote the article. YK provided critical revisions and final approval and had overall responsibility for the article.

### Conflict of interest statement

The authors declare that the research was conducted in the absence of any commercial or financial relationships that could be construed as a potential conflict of interest.

## Abbreviations

3-NPA: 3-nitropropionic acid
6-OHDA: 6-hydroxydopamine
AD: Alzheimer's disease
AIMs: abnormal involuntary movements
ALS: amyotrophic lateral sclerosis
AP-1: activator protein-1
APP: amyloid precursor protein
Akt: protein kinase B
BAD: Bcl2-associated agonist of cell death
BAK: Bcl2-antagonist/killer
Bax: Bcl-2–associated X protein
Bcl: B-cell lymphoma
BDNF: brain derived neurotrophic factor
CAT: catalase
Cdk: cyclin dependent kinase
DOPAC: dihydroxyphenyl acetic acid
ERK: extracellular signal-regulated kinase
FOXO: forkhead box class O
GSH: glutathione peroxidase
GSK: glycogen synthase kinase
HD: Huntington's disease
IMPase: inositol monophosphatase
IP3: inositol 1,4,5-trisphosphate
JNK: Jun N-terminal kinase
MCI: mild cognitive impairment
MDM: murine double minute
MEF: myocyte enhancement factor
MPTP: 1-methyl-4-phenyl-1,2,3,6-tetrahydropyridine
NPCs: neural progenitor cells
NT-3: neurotrophin 3
NT-4: neurotrophin 4
PD: Parkinson's disease
SNpc: substantia nigra pars compacta
SOD: superoxide dismutase
Tcf: T-cell factor
TH: tyrosine hydroxylase
TrkA: receptor tyrosine kinase A
TrkB: receptor tyrosine kinase B
TrkC: receptor tyrosine kinase C
VPA: valproic acid.
